# A eugenia no eixo luso-brasileiro: as cartas entre Renato Kehl e Mendes Correia

**DOI:** 10.1590/S0104-59702026000100014

**Published:** 2026-06-01

**Authors:** Daniel Florence Giesbrecht

**Affiliations:** i Investigador, Centro de Estudos Interdisciplinares/Universidade de Coimbra; professor, Faculdade de Letras/Universidade de Coimbra. Coimbra – Portugal. daniel.giesbrecht@uc.pt

**Keywords:** Eugenia atlântica, Epistolografia científica, Capital simbólico, História intelectual, Circulação transnacional de saberes, Atlantic eugenics, Scientific letters, Symbolic capital, Intellectual history, Transnational circulation of knowledge

## Abstract

Este estudo analisa, sob uma perspectiva crítica da história intelectual, o intercâmbio epistolar entre Renato Ferraz Kehl e António Mendes Correia, protagonistas dos discursos eugênicos no Brasil e em Portugal. As cartas são tratadas como dispositivos de consagração científica, revelando um campo transatlântico de trocas assimétricas marcadas por disputas de prestígio, estratégias editoriais e autoridade epistêmica. Com base nas categorias de *habitus* e capital simbólico (Bourdieu), argumenta-se que essas correspondências funcionam como artefatos performativos da racionalidade eugênica, situando-se entre o íntimo e o institucional. A análise ilumina conteúdos explícitos, mas também silêncios e frustrações que permeiam essa relação, revelando como a eugenia foi negociada e instrumentalizada no contexto atlântico luso-brasileiro.

## Abstract (en)

This study uses a critical intellectual history perspective to examine letters between Renato Ferraz Kehl and Antonio Mendes Correia, who were active in eugenic discourse in Brazil and Portugal. This correspondence serves as a vehicle of scientific consecration, revealing a transatlantic field of asymmetric exchanges marked by disputes for prestige, editorial strategies and epistemic authority. Based on the categories of habitus and symbolic capital, the letters are seen as performative artifacts of eugenic rationality, located between the intimate and the institutional. Our analysis reveals not only the contents but also the silences and frustrations within this relationship, showing how eugenics was negotiated and instrumentalized in the Atlantic Luso-Brazilian Atlantic context.

A eugenia, nas primeiras décadas do século XX, não se afirmou apenas como doutrina científica, mas como engrenagem de um imaginário civilizacional que buscava fundar novas ordens morais, biológicas e estatais, pautado pelas premissas da modernidade (Bauman, 1999). Ancorada em saberes médicos, pedagógicos e antropológicos, a eugenia se instituiu como projeto de regeneração coletiva e aperfeiçoamento racial, assumindo múltiplas feições em diferentes geografias – do higienismo urbano às políticas populacionais (Carvalho, 2022). Em contextos como o de Brasil e Portugal, marcados por um histórico de relação colonial e ansiedades quanto à identidade nacional, a eugenia foi apropriada e ressignificada de modo singular, isto é, um programa científico adaptado às realidades raciais e culturais específicas, ainda que permeado por referências epistemológicas do norte europeu (Cleminson, 2014; Souza, 2016).

O presente estudo (em andamento) propõe-se a explorar uma dimensão ainda marginal na historiografia da eugenia atlântica: as trocas epistolares entre Renato Ferraz Kehl – fundador da Sociedade Eugênica de São Paulo (1918), primeira instituição do gênero na América Latina, e principal expoente do movimento eugênico brasileiro – e António Mendes Correia, catedrático da Universidade do Porto e figura central da antropologia portuguesa (Souza, 2019; Matos, 2023; Giesbrecht, 2024). As principais fontes consultadas para essa investigação foram localizadas em arquivos no Brasil e em Portugal, sobretudo no Centro de Documentação e História da Saúde (Fundo Renato Kehl) da Fundação Oswaldo Cruz, no Rio de Janeiro, e no Centro de Memória (Fundo Santos Júnior) da Vila de Monte de Moncorvo.

Ao considerar essas cartas como dispositivos epistemológicos e políticos – mais do que apenas registros pessoais –, busca-se evidenciar os modos pelos quais o saber eugênico era performado, circulado e institucionalizado no eixo luso-brasileiro. Para tanto, mobilizam-se os instrumentos analíticos propostos por Pierre Bourdieu (1989, 2003) – notadamente *habitus* e capital simbólico – com o intuito de compreender como tais agentes posicionaram-se, discursivamente, em uma cartografia atlântica do saber eugênico. O conceito de *habitus* refere-se a esquemas de percepção e ação socialmente estruturados que moldam condutas, expectativas e estratégias no interior de um campo. Utiliza-se o conceito de “capital simbólico” para evidenciar como prestígio, autoridade e legitimidade operam como formas de poder reconhecidas socialmente. A investigação inscreve-se na tradição da história intelectual crítica, examinando não somente o conteúdo das trocas, mas sobretudo os dispositivos retóricos e as estruturas de poder que lhes conferiam eficácia simbólica (Sirinelli, 2003).

As cartas trocadas entre Kehl e Mendes Correia não devem ser compreendidas como meros veículos de cortesia intelectual ou atualizações bibliográficas. Elas operam como instrumentos de inscrição simbólica, cujas finalidades extrapolam a comunicação interpessoal e se enraízam em estratégias de legitimação científica, consagração institucional e difusão editorial. Tais documentos revelam que, sob a aparência do diálogo amistoso, se desenrola uma arquitetura relacional voltada para a produção de prestígio e a circulação de autoridade científica no espaço atlântico (Bourdieu, 2003).

Logo nas primeiras missivas, identificam-se gestos inequívocos de cooptação simbólica. Mendes Correia solicita a intermediação de Kehl junto a editoras e livrarias brasileiras para a difusão de suas obras antropológicas, lamentando, não sem ressentimento, o fraco impacto editorial de seus livros em território brasileiro. Em carta de 16 de dezembro de 1925, afirma ter “relações mais remuneradoras na Argentina do que no Brasil”, sublinhando a assimetria na recepção de sua produção intelectual (Correia, 16 dez. 1925). Kehl, por sua vez, retribui o pedido com envio de exemplares do livro *Bíblia da saúde*, o qual é prontamente elogiado por Correia (12 jul. 1926) como “obra de apostolado e vigor pedagógico”. Não se trata de um altruísmo desinteressado: o elogio atravessa um campo de expectativas mútuas. Mendes Correia compromete-se a resenhar *Bíblia da saúde* na imprensa portuguesa – inicialmente no jornal *O Primeiro de Janeiro*, de ampla circulação e prestígio no Porto –, mas opta por fazê-lo na revista da Sociedade Portuguesa de Antropologia e Etnologia (Spae), da qual era diretor (Correia, 1926). Ao assim proceder, inseriu o brasileiro na esfera intelectual especializada lusitana, conferindo-lhe legitimidade como autoridade no domínio do higienismo e da eugenia. Esse circuito, como propõe Heilbron (1999), pode ser entendido como um sistema de trocas assimétricas em que obras, elogios, colaborações e referências funcionam como formas de capital simbólico. A rede entre os dois autores articula-se como uma zona de interdependência estratégica: Correia oferece institucionalidade e tradição acadêmica; Kehl fornece acesso a um público leitor amplo e um mercado editorial em expansão (Bragança, Abreu, 2010).

Na prática, as cartas funcionam como dispositivos operacionais de pertencimento e consagração: as assinaturas, os timbres, os modos de enunciação e os circuitos por onde transitam revelam um projeto de inserção simultânea em dois campos científicos em constituição. A partir da epístola, ambos constroem sua autoridade discursiva não apenas perante o outro, mas diante de um público ampliado, ainda que invisível, composto por leitores potenciais, mediadores editoriais e instituições legitimadoras. Além disso, as correspondências registram não somente intenções, mas também silêncios. Em alguns trechos, percebe-se a frustração de Kehl com a falta de retorno de editoras e a hesitação de Correia diante da ausência de resposta de instituições brasileiras. Lapsos, ausências e incômodos são parte do que as cartas produzem: elas performam tanto o desejo de conexão quanto os limites estruturais da própria circulação atlântica de saberes. E é nesse jogo de presença e ausência, expectativa e frustração, que os artefatos epistolares revelam sua potência como fontes históricas – não como espelhos da realidade, mas como operadores da experiência científica moderna (Farge, 1989).

O intercâmbio entre Kehl e Mendes Correia deve ser situado em um contexto histórico mais amplo, no qual a eugenia funcionava como matriz racionalizadora de projetos imperiais, programas sanitários e ambições nacionais. No Brasil, a institucionalização da eugenia esteve profundamente vinculada à agenda republicana de modernização, ao passo que, em Portugal, se articulavam às necessidades simbólicas de um império em declínio, cuja sobrevivência passava por novas formas de justificação científica e antropológica (Pimentel, 1998; Stepan, 2005).

A correspondência evidencia como ambos os autores utilizavam a eugenia como linguagem comum para interpretar os destinos raciais de seus respectivos países. Em uma carta de 1932, Mendes Correia convida Kehl para proferir uma conferência sobre política eugênica no Porto, reconhecendo-o como “referência incontornável” no que toca ao aprimoramento da raça latino-americana (Correia, 18 jun. 1932). Kehl (25 jul. 1932) acolheu com manifesta exultação o convite que lhe foi endereçado e, sem demora, remeteu-lhe o ensaio intitulado “Política eugênica”, no qual condensava os principais postulados doutrinários que vinham norteando sua militância eugênica no Brasil. A conferência, concebida desde o início como peça de difusão pública e legitimadora de seu prestígio internacional, seria proferida em sessão solene da Spae em 24 de outubro de 1932, por ocasião da sua visita a terras portuguesas. Ciente do valor estratégico da ocasião e do prestígio que ela emprestaria ao seu interlocutor, Mendes Correia fez questão de assegurar que o referido manuscrito fosse publicado, na íntegra, no órgão oficial da Spae, chancelando-o como contribuição de vulto para os debates eugênicos em Portugal (Kehl, 1933). O texto, carregado de referências à hereditariedade, ao casamento racional e ao valor biológico da infância, foi recebido como manifesto técnico e moral em favor de uma pedagogia da regeneração. Neste último tópico, Kehl advogava que a infância deveria ser objeto de um disciplinamento rigoroso – físico, moral e intelectual – capaz de assegurar não apenas a reprodução de qualidades herdadas, mas sobretudo o desenvolvimento de um corpo social higienicamente conduzido desde os primeiros anos. A criança, convertida em capital biológico da nação, deveria ser educada em harmonia com os princípios da medicina preventiva e da seleção eugênica, sob pena de comprometer o porvir da raça (Kehl, 1933, p.7-8).

A circulação de ideias, textos e livros entre os dois continentes revela uma operação simbólica mais profunda: ao exportar suas publicações para o Brasil, Mendes Correia buscava reposicionar a antropologia portuguesa no cenário das ciências raciais modernas. Kehl (26 jul. 1930), por sua vez, apresentava o Brasil como um “grande laboratório etnológico” e espaço de aplicação da eugenia, numa tentativa de inscrever-se nos circuitos científicos internacionais – sem deixar de demarcar sua “originalidade tropical” (Kehl, 26 jul. 1930). O projeto de ambos, portanto, não se esgotava na circulação de conteúdos, mas implicava uma disputa por centralidade epistêmica no campo eugênico atlântico, na qual o saber europeu ainda gozava de autoridade, mas já se via confrontado por experiências oriundas da periferia científica.

Nesse processo, as correspondências até agora investigadas não somente revelam o diálogo intelectual consolidado entre Kehl e Mendes Correia, mas também permitem identificar nomes até então pouco estudados, ampliando o escopo de análise para atores menos visíveis ou mesmo esquecidos (Figura 1).

**Figura 1:**
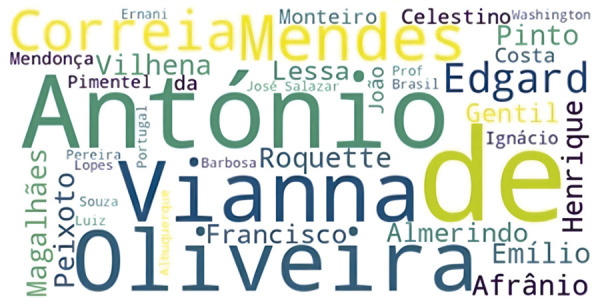
: Representação visual da frequência e relevância de personagens a partir da análise de correspondências epistolares (Fonte: elaborado pelo autor utilizando o *software* Python e a biblioteca WordCloud)

Tal cartografia relacional não somente amplia o espectro dos agentes envolvidos na produção e circulação do saber eugênico, mas também ilumina as engrenagens institucionais, os vínculos de prestígio e os mecanismos de legitimação que conferiam densidade às práticas biopolíticas do período (Foucault, 1997). Médicos portugueses como Almerindo Lessa, pioneiro nos estudos de hemoterapia; Enrique de Vilhena, renomado anatomista e prolífico divulgador científico; e Antônio Emílio de Magalhães, figura de proa na fundação da Liga Portuguesa de Profilaxia Social, mantinham interlocução frequente com Kehl, participando do mesmo circuito de consagração e intercâmbio. Do lado brasileiro, nomes como Afrânio Peixoto – médico de prestígio e ensaísta amplamente reconhecido – e o historiador Pedro Calmon – cuja atuação política e acadêmica o aproximava das elites letradas luso-brasileiras – apareciam associados a Mendes Correia, compondo com ele uma constelação intelectual que articulava medicina, história, pedagogia e antropologia sob a égide do discurso eugênico.

O gesto epistolar, assim, compõe uma cena complexa em que se entrelaçam confiança, cálculo, diplomacia e ambição. A carta assume o papel de mediação entre mundos, transpondo fronteiras disciplinares e nacionais (Saunier, 2013). Por ela, passam não apenas ideias, mas também expectativas, ressentimentos, hierarquias e projetos. E é justamente nesse entrelugar – entre o íntimo e o institucional, o protocolar e o estratégico – que a epístola adquire sua força histórica: como artefato que simultaneamente documenta e constitui o campo científico entre a geografia dos atores envolvidos (Chartier, 1990). Outro aspecto relevante a ser considerado é que a cooperação entre Renato Kehl e António Mendes Correia, apesar de ostentar um discurso de fraternidade lusófona, é marcada por assimetrias estruturais que reproduzem lógicas coloniais no campo científico atlântico. A análise das cartas revela que a relação entre ambos se constrói sobre um delicado equilíbrio entre deferência e reivindicação, em que o Brasil oscila entre ser receptor entusiástico do saber europeu e produtor de uma ciência localizada, ansiosa por reconhecimento internacional.

Mendes Correia, frequentemente, assume uma posição que ressoa com a autoridade metropolitana. Solicita informações, fotografias e dados sobre os “tipos humanos” do Brasil, sugerindo uma taxonomia racial que se pretendia sistematizar a partir da lógica da antropologia física clássica. Kehl, por sua vez, apresenta-se como colaborador diligente, mas reivindica a originalidade e a relevância das experiências eugênicas brasileiras, sobretudo aquelas voltadas para a pedagogia social, a medicina social e a racionalização dos casamentos. Esses movimentos indicam que, apesar da colaboração, o fluxo simbólico permanece assimétrico (Heilbron, 1999). O saber português, mesmo sem a força institucional de outros centros europeus, ancora-se em sua tradição para legitimar-se perante o Brasil. O saber brasileiro, por sua vez, busca no prestígio europeu uma chancela que lhe permita transitar nos circuitos internacionais. A relação, portanto, está marcada por um desequilíbrio epistemológico, em que os critérios de validação continuam a ser definidos a partir do centro (Heilbron, 1999).

## Considerações finais

A análise da correspondência entre Renato Kehl e António Mendes Correia permite iluminar as dinâmicas profundas que estruturaram o campo eugênico luso-brasileiro entre as décadas de 1920 e 1930. Muito além de uma relação epistolar pontual, as cartas revelam um sistema de produção, legitimação e circulação de saberes no espaço atlântico marcado por alianças estratégicas, disputas simbólicas e persistentes hierarquias coloniais. Kehl e Correia não somente trocaram livros e elogios: construíram, por meio da escrita, um campo de consagração mútua que articulava prestígio institucional, circulação editorial e pertencimento a uma comunidade científica transnacional (Latour, 2006). Ao atuarem como mediadores entre metrópole e ex-colônia, ambos inscreveram-se – ainda que em posições desiguais – em uma cartografia de saber que buscava, a um só tempo, afirmar singularidades nacionais e submeter-se aos cânones europeus da ciência moderna.

A epístola, nesse contexto, não é apenas meio de comunicação, mas tecnologia de inscrição simbólica, capaz de produzir pertencimento, performar autoridade e constituir redes (Barnes, 1987). Por meio dela, se delineia um projeto de ciência racializada que, ao mesmo tempo que se quer tropical e moderno, permanece ancorado em estruturas hierárquicas herdadas do passado colonial. A carta transforma-se, assim, em testemunho e vetor: testemunho de uma relação científica marcada por afetos, frustrações e ambições; vetor de uma política do saber que define quem pode falar, de onde e com que legitimidade. Ao trazer à luz esse *corpus* epistolar, a investigação tem em vista contribuir para uma compreensão mais complexa das formas pelas quais a eugenia foi negociada, institucionalizada e performada no eixo luso-brasileiro. Reafirma, também, a importância da história intelectual crítica como via para desnaturalizar narrativas lineares de progresso científico e recolocar no centro da análise os dispositivos relacionais, discursivos e simbólicos que constituem a ciência como prática social e política.

## Data Availability

Não estão em repositório.
